# The Beneficial Effects of Two Polysaccharide Fractions from *Sargassum fusiform* against Diabetes Mellitus Accompanied by Dyslipidemia in Rats and Their Underlying Mechanisms

**DOI:** 10.3390/foods11101416

**Published:** 2022-05-13

**Authors:** Rui-Bo Jia, Juan Wu, Donghui Luo, Lianzhu Lin, Chong Chen, Chuqiao Xiao, Mouming Zhao

**Affiliations:** 1 Chaozhou Branch of Chemistry and Chemical Engineering Guangdong Laboratory, Chaozhou 521000, China; jiaruibo@hotmail.com (R.-B.J.); luodonghui@gdou.edu.cn (D.L.); felzlin@scut.edu.cn (L.L.); 2 School of Food Science and Engineering, South China University of Technology, Guangzhou 510640, China; wuyajuan512@163.com; 3 School of Food Science and Engineering, Guangdong Ocean University, Yangjiang 529500, China; 4 Hainan Key Laboratory of Storage and Processing of Fruits and Vegetables, Agricultural Products Processing Research Institute, Chinese Academy of Tropical Agricultural Sciences, Zhanjiang 524001, China; chenchong_cristina@163.com

**Keywords:** *Sargassum fusiform* polysaccharides, antidiabetic activities, underlying mechanisms

## Abstract

The current study aimed to assess the anti-diabetic effects and potential mechanisms of two *Sargassum fusiform* polysaccharide fractions (SFPs, named SFP-1 and SFP-2). The carbohydrate-loading experiment revealed that SFP-2 could control postprandial hyperglycemia by inhibiting the activity of digestive enzymes in rats. The analysis of diabetic symptoms and serum profiles indicated that SFPs could mitigate diabetes accompanied by dyslipidemia, and SFP-2 showed better regulatory effects on body weight, food intake and the levels of total cholesterol (TC), triglycerides (TG), low density lipoprotein-cholesterol (LDL-C) and free fatty acid (FFA) in diabetic rats. Intestinal bacterial analysis showed that SFP treatment could reshape the gut flora of diabetic rats, and SFP-2 possessed a greater regulatory effect on the growth of *Lactobacillus* and *Blautia* than SFP-1. RT-qPCR analysis revealed that SFPs could regulate the genes involved in the absorption and utilization of blood glucose, hepatic glucose production and lipid metabolism, and the effects of SFP-2 on the relative expressions of Protein kinase B (Akt), Glucose-6-phosphatase (G-6-Pase), Glucose transporter 2 (GLUT2), AMP-activated protein kinase-*α* (AMPK*α*), Peroxisome proliferator-activated receptor *γ* (PPAR*γ*) and Cholesterol 7-alpha hydroxylase (CYP7A1) were greater than SFP-1. All above results indicated that SFPs could be exploited as functional foods or pharmaceutical supplements for the treatment of diabetes and its complications.

## 1. Introduction

Type 2 diabetes mellitus (T2DM) is a chronic metabolic disease caused principally by insulin dysfunction [1]. Simultaneously, T2DM is frequently accompanied by hyperlipidemia, retinopathy, nephropathy, cardiovascular disease, etc., which can further lead to death and disability [2]. According to statistics from the International Diabetes Federation (2019), there are about 463 million diabetic patients (20–79 years old) in the world, about 4.2 million deaths are linked to diabetes or its complications, and the total medical cost of diabetes is as high as 760.3 billion; all of these could be a huge public health challenge and economic burden [3]. Although there are many drugs applied in diabetes treatment, all have certain side effects, such as liver and kidney damage, gastrointestinal side effects, hypoglycemia, etc. [4,5]. Therefore, developing novel bioactive ingredients and exploring the hypoglycemic mechanisms are particularly significant for the prevention and adjuvant treatment of T2DM.

Polysaccharides have gained increasing attention in the field of medicine and functional foods due to their non-toxic characteristics and multiple biological activities [6]. A myriad of evidence has demonstrated that polysaccharides from natural sources such as *Ganoderma lucidum*, *Dendrobium huoshanense*, *Abelmoschus esculentus*, *Ophiopogon japonicus* and *Grifola frondosa* possess potential hypoglycemic and hypolipidemic activities [4,7,8,9,10,11]. These polysaccharides could affect the development and progression of diabetes through multiple approaches, such as inhibiting carbohydrate hydrolase activities, repairing pancreatic *β* cells, inhibiting gluconeogenesis and improving insulin resistance [12,13]. Nowadays, accumulating evidence has also suggested that the intestinal microbiome plays a fundamental role in metabolic syndromes such as obesity, diabetes and cardiovascular disease [14], while diabetics are characterized by a certain degree of gut microbial dysbiosis [15]. It is generally acknowledged that polysaccharides, a class of superior prebiotics, could be hydrolyzed and fermented by intestinal flora to produce monosaccharides and short-chain fatty acids (SCFAs), which could affect host health [16,17,18,19].

*Sargassum fusiform*, also known as “longevity vegetable”, a kind of brown algae with potential development value, is widely distributed in China, Japan and Korea, and it has been applied as a Chinese herbal medicine for thousands of years [20]. Researchers found that *Sargassum fusiforme* polysaccharides possessed multiple physiological functions, such as reducing cholesterol, antitumor activity, adjusting immunity and improving diabetes [6]. Previously, we analyzed the chemical compositions and structural characteristics of two polysaccharide fractions from *Sargassum fusiforme* and confirmed their hypoglycemic activity through oral administration in rats for 5 weeks. However, their anti-diabetic approaches and underlying molecular mechanisms were not clarified [21]. In the current research, a T2DM animal model was set up, using a high-fat diet and streptozotocin (STZ) injection, and the anti-diabetic activities of two polysaccharide fractions were further assessed for 8 weeks. Meanwhile, we aimed to determine their anti-diabetic mechanisms from the perspectives of inhibiting blood glucose elevation caused by eating, remodeling intestinal flora, activating insulin signaling pathways, and suppressing gluconeogenesis and lipid accumulation.

## 2. Materials and Methods

### 2.1. Material and Chemicals

*Sargassum fusiforme* was purchased from Qingdao Fuxingxiang Import & Export Co., Ltd. (Shandong, China). Raw materials were washed with running tap water, then soaked in industrial alcohol for 24 h. The dried powder was acquired and stored at room temperature for further use. The voucher specimen (FSLs-045-2021) was deposited with the Food Science Laboratory of Chaozhou Branch of Chemistry and Chemical Engineering Guangdong Laboratory (Chaozhou, China).

Pectinase, papain and cellulase were purchased from Solarbio Science & Technology Co., Ltd., Beijing, China. Acarbose was produced at Luye Pharmaceutical Co., Ltd., Sichuan, China. Streptozocin (STZ) was obtained from Sigma-Aldrich (Burlington, MA, USA). Other reagents used in this paper were analytical grade.

### 2.2. Preparation of SFPs

SFP-1 and SFP-2 were prepared by referring to our previous report [21]. Before extracting, the *Sargassum fusiforme* powder was pretreated using dual enzyme (1% pectinase, 1% cellulase, pH = 4.5, 50 °C) for 60 min, followed by ultrasonic treatment at a power of 400 W. Subsequently, the mixture was extracted with hot water at 90 °C for 2 h, then cooled and centrifuged (2000× *g*, 20 min). The supernatant was supplemented with 1% papain at 55 °C for 1 h, then heated by boiling water for 5 min and centrifuged (2000× *g*, 20 min). The supernatant was ultrafiltered by an ultrafiltration membrane (3 kDa) cut-off for 5 times and concentrated using a vacuum rotary evaporator. The concentrate was precipitated with 80% (*v*/*v*) ethanol at 4 °C for 24 h. After centrifugation, the precipitate was collected, and the protein was removed by the Sevag method [22]. Finally, the sample solution was ultrafiltered with 10 kDa cut-off for 5 times. The macromolecular portion and small molecule portion were collected and lyophilized, and named SFP-1 and SFP-2, respectively.

In our previous findings, the characteristics of SFP-1 and SFP-2 were reported in detail [21]. Concretely, the total carbohydrates of SFP-1 and SFP-2 was about 69% and 84%, the uronic acid was about 10% and 38%, the sulfate was about 10% and 6%, respectively. SFP-1 and SFP-2 possessed analogous monosaccharides, but the molar ratios were different; SFP-1 was principally composed of 80.04% glucose, 5.71% mannose, 4.03% galactose and 4.93% glucuronic acid, and SFP-2 consisted of chiefly 41.22% fucose, 19.27% galactose, 16.79% mannose and 13.40% glucuronic acid. The molecular weight (Mw) analysis revealed that SFP-1 was found to consist of two peaks with an Mw of 8.47 (69.42%) and 4.33 kDa (30.58%), and SFP-2 was found to be split up into two fractions (2 peaks) with an Mw of 84.99 (92.22%) and 14.33 (7.78%) kDa.

### 2.3. Animal Experiment

Five-week-old male SD rats (50 rats) were purchased from the Guangdong Medical Laboratory Animal Center (Foshan, China). All rats were raised in a stable sterile environment (24–26 °C, 50–60% relative humidity) with a light/dark cycle. The experiments were carried out in the order of Experiment 1, Experiment 2 and Experiment 3, and each experiment was performed after seven days of adaptive feeding.

#### 2.3.1. Experiment I

Thirty-two SD rats were stochastically divided into 4 groups of eight, as follows: (1) Control group (treated with 4 g/kg starch); (2) Acarbose group (treated conjointly with 30 mg/kg acarbose and 4 g/kg starch); (3) SFP-1 group (treated conjointly with 4 g/kg starch and 400 mg/kg SFP-1); (4) SFP-2 group (treated conjointly with 4 g/kg starch and 400 mg/kg SFP-2). Rats were handled according to the experimental design, and blood glucose levels were measured at 0, 30, 60, 90 and 120 min.

#### 2.3.2. Experiment II

Twenty-four SD animals were randomly split into 3 groups (*n* = 8): (1) Control group (treated conjointly with 2 g/kg glucose); (2) SFP-1 group (treated conjointly with 2 g/kg glucose and 400 mg/kg SFP-1); (3) SFP-2 group (treated conjointly with 2 g/kg glucose and 400 mg/kg SFP-2). Rats were handled according to the set experiment group and dose, and blood glucose levels were measured at 0, 15, 30, 60 and 90 min.

#### 2.3.3. Experiment III

All SD rats were randomly divided into 2 groups: (1) NFD group (8 rats, fed with conventional diets); (2) T2D group (42 rats, fed with high-fat diets). After 30 days, T2D group rats were intraperitoneally injected with fresh streptozotocin solution (25 mg/kg, 3 days), and NFD group animals were injected with saline. One week later, the fasting blood glucose levels were measured, and animals with blood glucose levels ≥11.1 mmol/L were identified as diabetic rats. Diabetic rats were randomly subdivided into the following groups (*n* = 8): (1) HFD group (gavaged with saline); (2) SFP-1 group (gavaged with 400 mg/kg/d body weight SFP-1); (3) SFP-2 group (gavaged with 400 mg/kg/d body weight SFP-2). The fasting blood glucose, body weight, food intake and water intake were recorded weekly from 0 weeks to 8 weeks. When the experiment was over, all rats were sacrificed using ether after fasting for 12 h. The whole blood was collected for detecting relevant indexes. The liver was isolated immediately and stored at −80 °C for gene expression analysis. Fecal samples were obtained and kept in liquid nitrogen for intestinal microflora composition and short-chain fatty acid content detection.

### 2.4. Oral Glucose Tolerance Test

All animals were orally supplemented with glucose solution (1.5 g/kg). The concentration of blood glucose was determined at 0, 30, 60, 90, 120 and 180 min using an Omron blood glucose tester (HGM-111), and the area under the curve (AUC) was assessed as follows:

AUC = (0.5 × GLU_1_ + GLU_2_ + GLU_3_ + GLU_4_ + GLU_5_ + 0.5 × GLU_6_) × 30

where GLU_1_, GLU_2_, GLU_3_, GLU_4_, GLU_5_ and GLU_6_ represent the blood glucose level at 0, 30, 60, 90, 120 and 180 min, respectively.

### 2.5. Biochemical Index Determination

The blood glucose was detected using an Omron blood glucose tester (Kyoto, Japan) (HGM-111). Relevant parameters, including glycosylated hemoglobin (HbA1c), insulin, triglycerides (TG), total cholesterol (TC), low density lipoprotein-cholesterol (LDL-C), high density lipoprotein-cholesterol (HDL-C), free fatty acid (FFA), total bile acid (TBA), alanine transaminase (ALT), aspartate transaminase (AST), creatinine (Cr), blood urea nitrogen (BUN), Interleukin-6 (IL-6), tumor necrosis factor-*α* (TNF-*α*), superoxide dismutase (SOD), glutathione peroxidase (GSH-Px) and malonic dialdehyde (MDA) were determined using assay kits (Jiancheng Biological Engineering Institute, Nanjing, China). In addition, the HOMA-IR was calculated according to the following formula:

HOMA-IR = the blood glucose level (mmol/L) × insulin (mIU/L)/22.5

### 2.6. Short Chain Fatty Acid Detection

Feces (20 mg) with anhydrous methanol (1 mL) was mixed using a vortex mixer, then placed at 4 °C for 24 h after ultrasound treatment (400 W) for 30 min. The supernatant was collected by centrifugation for further analysis. The SCFA concentrations were assessed by referring to already reported methods based on gas chromatography [23].

### 2.7. 16S rRNA Gene Sequencing Analysis

Genome DNA was extracted using a commercial kit and referring to the manufacturer’s instructions. The V3–V4 region was amplified by primer 338F (5′-CCTACGGRRBGCASCAGKVRVGAAT-3′) and primer 806R (5′-GGACTACNVGGGTWTCTA ATCC-3′). PCR amplicons were barcoded, quantified and sequenced after being purified and recovered. The gut flora analysis was carried out at Shanghai Jingzhou Gene Technology Co., Ltd. (Shanghai, China). The overall changes of intestinal flora were presented using SIMCA 14.1 (Umetrics, Umea, Sweden). Difference analysis of different groups at the genus level was screened by linear discriminant analysis (LDA) effect size (LEfSe) algorithm through the Huttenhower Lab Galaxy Server (http://huttenhower.sph.harvard.Edu/lefse/ accessed on: 14 January 2022). The potential relationship between the gut flora regulation and hypoglycemic effects was revealed by correlation analysis using R software (Ver. 3.3.3) and Cytoscape (Ver. 3.6.0).

### 2.8. Quantitative Reverse Transcription PCR

The total RNA of the livers from rats was extracted and purified using a kit (Takara, Japan), and the cDNA was synthesized using a reverse transcription kit (Takara, Japan). PCR was performed using a CFX96 Real-Time PCR System (Bio-Rad, Hercules, CA, USA) with the SYBR^®^ Premix Ex Taq™ (Takara, Japan), and all primer sequences are listed in Table 1. The mRNA expression was calculated using the formula of 2^−ΔΔCt^ after normalized to β-actin.

### 2.9. Statistical Analysis

All of the data were displayed as mean ± SD. Statistical difference between groups was analyzed by Duncan’s test and one-way analysis of variance (ANOVA) test performed by SPSS 20 software. *p* < 0.05 was considered as the statistically significant difference.

## 3. Results

### 3.1. Effects of SFPs on Carbohydrate Loading Capacity in Rats

**Figure 1:**
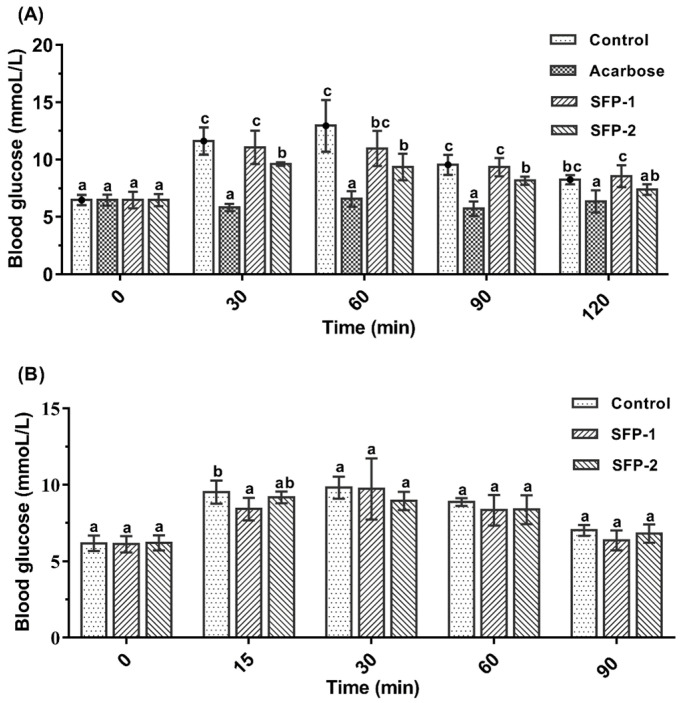
Effects of SFPs on blood glucose after starch loading (**A**) and glucose loading (**B**) in rats. The different letters represent significant differences (*p* < 0.05).

Inhibiting the activity of carbohydrate decomposition enzymes in the process of food digestion, and delaying the passage of glucose through the cells of the small intestine can effectively delay postprandial hyperglycemia [24]. The carbohydrate-loading experiment was designed to assess the efficacy of SFPs in controlling postprandial blood glucose fluctuations in rats. As shown in Figure 1A, the blood glucose level was significantly reduced in Acarbose and SFP-2 groups with respect to the control group (*p* < 0.05). However, there was no significant variation in blood glucose level between the control group and SFP-1 group during the whole experiment. Therefore, it could be concluded that SFP-2 could inhibit the blood glucose increase caused by eating. The glucose loading test results are shown in Figure 1B. Compared with the control group, the blood glucose levels of the SFP-1 group were significantly reduced at 15 min (*p* < 0.05). Furthermore, the blood glucose of rats in all groups showed no significant differences within 30 to 90 min (*p* > 0.05). These results show that SFP-2 could restrain postprandial hyperglycemia by inhibiting carbohydrate hydrolase activity.

### 3.2. Hypoglycemic Activity of SFPs in Diabetic Rats

**Figure 2:**
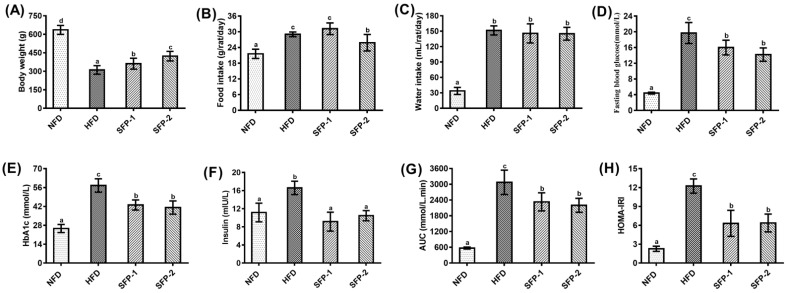
Effects of SFPs on body weight (**A**), food intake (**B**), water intake (**C**), fasting blood glucose level (**D**), HbA1c (**E**), insulin (**F**), AUC (**G**) and HOMA-IRI (**H**) in diabetic rats. The different letters represent significant difference (*p* < 0.05).

As shown in Figure 2, rats from the HFD group showed typical diabetic symptoms (loss of body weight, increase of food and water intake), hyperglycemia (high fasting blood glucose and glycosylated hemoglobin), impaired glucose tolerance and insulin resistance compared with rats from the NFD group. In contrast, body weight loss and food intake increase were attenuated after SFP-2 intervention for 8 weeks, whereas SFP-1 supplementation only effectively prevented body weight loss (Figure 2A,B). There was no statistical difference in each group regarding water intake of rats, except for the NFD group (Figure 2C). In addition, in comparison with the rats of the HFD group, SFPs supplementation could obviously reduce the fasting blood glucose (Figure 2D), glycosylated hemoglobin (Figure 2E) and insulin (Figure 2F) levels as well as lower AUC (Figure 2G) and HOMA-IRI (Figure 2H) values. The results showed that both SFP-1 and SFP-2 possessed significant hypoglycemic activity, and SFP-2 was superior to SFP-1 in preventing loss of body weight and increase of food intake.

### 3.3. Effects of SFPs on Serum Profiles in Diabetic Rats

**Figure 3:**
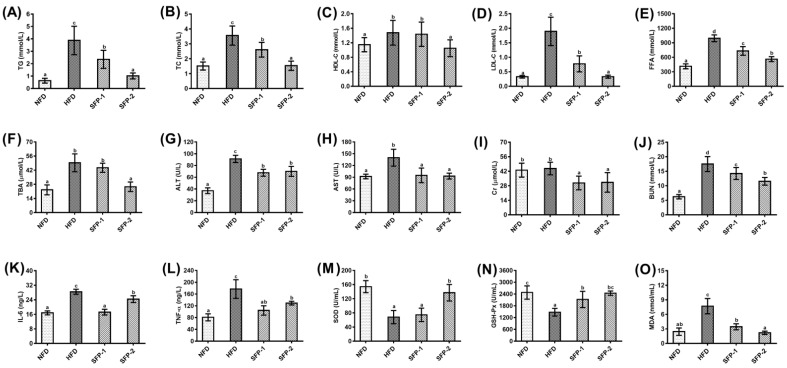
Effects of SFPs on serum profiles in diabetic rats: TG (**A**), TC (**B**), HDL-C (**C**), LDL-C (**D**), FFA (**E**), TBA (**F**), ALT (**G**), AST (**H**), Cr (**I**), BUN (**J**), IL-6 (**K**), TNF-*α* (**L**), SOD (**M**), GSH-Px (**N**) and MDA (**O**). The different letters represent significant difference (*p* < 0.05).

Accumulating data have suggested that diabetes mellitus is associated with a high probability of complications, and the deterioration associated with such complications exerts greater harm to a patient’s health than diabetes alone [25]. As a consequence, reducing complications is of momentous significance for diabetes treatment. Figure 3A–E displays the serum lipid levels of rats in each group, and the results show that lipid indicators such as TC, TG, LDL-C and FFA were significantly increased after high-fat diet and STZ induction (*p* < 0.05). SFP treatment could markedly reduce the levels of these indicators to different degrees (*p* < 0.05). In particular, SFP-2 showed better improvement of lipid metabolism than SFP-1. In comparison to the HFD group, oral administration of SFPs did not increase the level of HDL-C, but HDL-C/LDL-C was increased after SFP treatment. As an effect of SFPs on liver and kidney function of diabetic rats (shown in Figure 3F–J), the serum concentrations of TBA, ALT, AST and BUN were markedly elevated in the HFD group when compared with the NFD group (*p* < 0.05). SFP administration lightened liver and kidney damage, which was manifested by a decrease in serum TBA, ALT, AST, Cr and BUN levels of rats from SFP-1 and SFP-2 groups in contrast to the HFD group (*p* < 0.05); SFP-2 was superior to SFP-1 in reducing the serum levels of TBA and BUN (*p* < 0.05). Serum concentrations of IL-6 and TNF-*α* are summarized in Figure K–L. Statistical difference was observed between the NFD group and HFD group (*p* < 0.05). As anticipated, IL-6 and TNF-*α* levels in SFP treatment groups were prominently lowered compared to the HFD group (*p* < 0.05). In addition, we also found that the effect of SFPs on oxidative stress levels in diabetic rats (see Figure 3M–O) indicated that the activities of SOD and GSH-Px in the HFD group were remarkably reduced, while MDA was observably higher than the NFD group (*p* < 0.05). It is worth noting that the change tendency of serum GSH-Px and MDA levels in diabetic rats were reversed by SFP intervention, and the serum SOD level of the SFP-2 group was significantly elevated compared with the HFD group (*p* < 0.05). To summarize, SFP supplementation markedly improved serum profiles, and SFP-2 treatment showed greater positive effects on some indexes, such as TC, TG, LDL-C, FFA, TBA, BUN, SOD and MDA, than SFP-1 treatment.

### 3.4. Effects of SFPs on SCFA Production Capacity in Diabetic Rats

**Figure 4:**
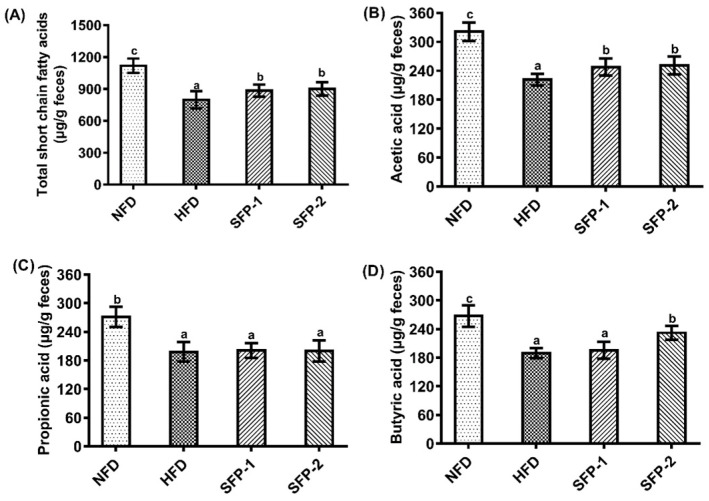
Effects of SFPs on production of short chain fatty acids in diabetic rats. Total short chain fatty acids (**A**), acetic acid (**B**), propionic acid (**C**) and butyrate (**D**). The different letters represent significant difference (*p* < 0.05).

The levels of SCFAs in the feces samples of rats in each group are shown in Figure 4. Figure 4A shows that the total SCFA concentration in the HFD group was prominently lower than the NFD group (*p* < 0.05). SFPs administration promoted the production of SCFAs, which may be due to the selective promotion of intestinal flora by SFPs. Figure 4B–D presents the levels of acetic acid, propionic acid and butyric acid, respectively. The evidence suggests that the acetic acid level of SFP treatment groups was significantly higher than the HFD group, and the butyric acid level of the SFP-2 group was significantly increased compared with the HFD group (*p* < 0.05). The data proved that SFP treatment might accelerate the conversion of indigestible carbohydrates into SCFAs, and the positive effect of SFP-2 on acetic acid and butyric acid producing bacteria was better than that of SFP-1.

### 3.5. Effects of SFPs on Gut Flora in Diabetic Rats

**Figure 5:**
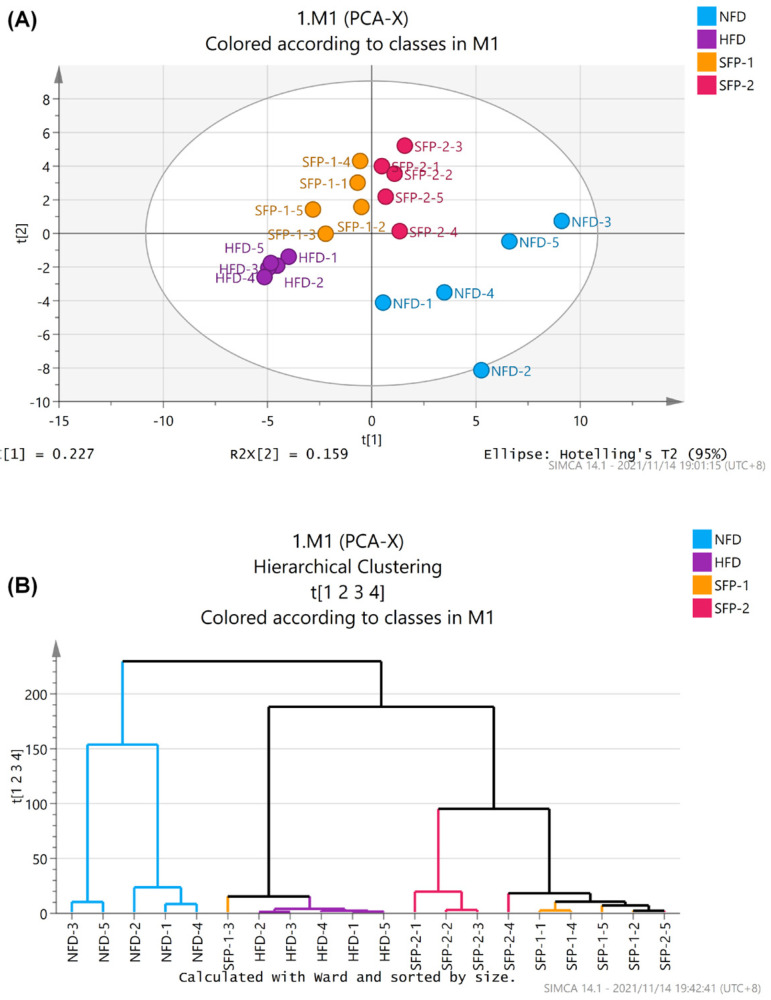
Effects of SFPs on gut microbiota in diabetic rats. Principal component analysis (**A**), hierarchical clustering tree analysis (**B**).

Principal component analysis (PCA) and cluster analysis were used to reveal the overall composition of gut microbiota. An apparent community separation was observed among the NFD group, HFD group and SFP administration groups (Figure 5A,B), indicating that the group differences in the flora structure was greater than individual differences.

**Figure 6:**
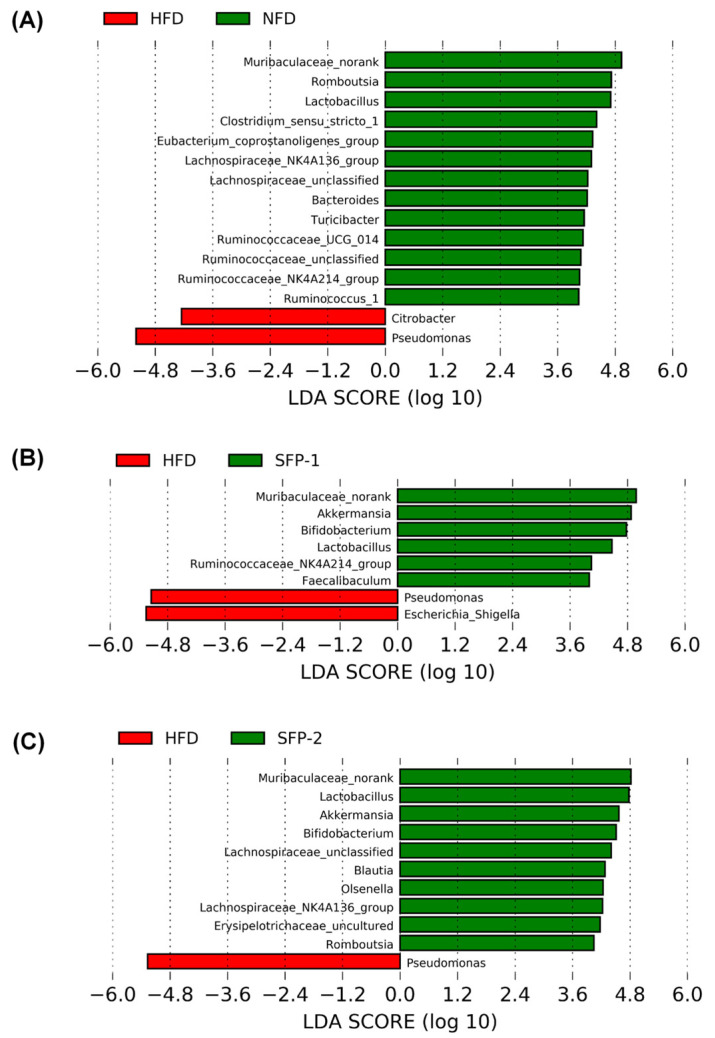
The differences of gut microbiota at the genus levels among groups. LEfSe comparison between HFD and NFD (**A**), LEfSe comparison between HFD and SFP-1 (**B**) and LEfSe comparison between HFD and SFP-2 (**C**). *p* < 0.05 and LDA score > 4.0 were considered statistically significant.

In addition, LDA score plot analysis among each group was further adopted to investigate the alterations in the gut microbiota composition at the genus levels. As displayed in Figure 6A, in comparison to the NFD group, the relative abundances of some genera, including *Muribaculaceae_norank*, *Clostridium*_sensu_stricto_1, *Eubacterium_coprostanoligenes*_group, *Lactobacillus*, *Romboutsia*, *Bacteroides*, *lachnospiraceae*_NK4A136_group, *Ruminococcus_*1, *Ruminococcaceae*_UCG-014 and *Ruminococcaceae*_NK4A214_group, were significantly reduced, whereas the amount of *Citrobacter* and *Pseudomonas* were obviously increased in the HFD group (*p* < 0.05, LDA SCORE > 4). The information from Figure 6B,C shows that SFP administration prominently increased the relative abundance of *Muribaculaceae_norank*, *Akkermansia*, *Bifidobacterium* and *Lactobacillus* and remarkably decreased the relative abundance of *Pseudomonas* in diabetic rats (*p* < 0.05, LDA SCORE > 4). In contrast, SFP-1 administration distinctly upgraded the relative abundance of *Ruminococcaceae*_NK4A214_group and *Faecalibaculum* and visibly lowered the relative abundance of *Escherichia-shigella* (*p* < 0.05, LDA SCORE > 4); however, SFP-2 significantly up-regulated the relative abundance of *Blautia*, *Olsenella*, *lachnospiraceae*_NK4A136_group and *Romboutsia* when compared to the HFD group. In addition, the relative abundance of *Lactobacillus* and *Blautia* in the SFP-2 group was significantly higher than in the SFP-1 group.

**Figure 7:**
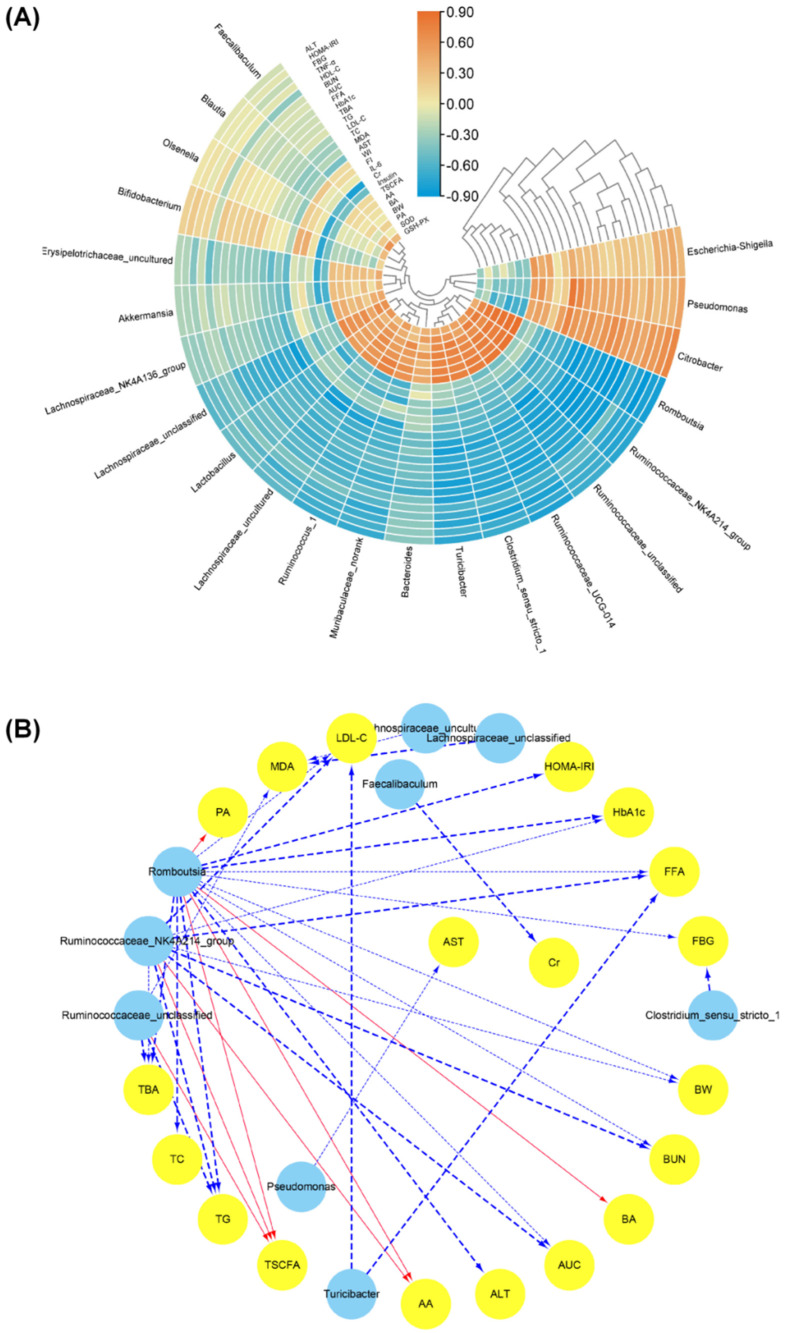
Spearman’s correlation between differential intestinal flora and diabetes-related indicators. Heatmap of Spearman’s correlation (**A**) and visualization of the correlation network (**B**). Note: the significant edges were drawn in the network using the Spearman correlation test (|r| > 0.8, FDR adjusted *p* < 0.01).

To further explore the influence of gut flora on the regulation of glucose and lipid metabolisms, the correlation analysis between the relative abundance of gut microbiota and diabetes-related parameters were calculated using Spearman correlation test (Figure 7A). The analysis result demonstrated that the abundance of *Clostridium*_sensu_stricto_1, *Eubacterium_coprostanoligenes*_group, *Lactobacillus*, *Muribaculaceae_norank*, *Romboutsia*, *Ruminococcaceae*_NK4A214_group, *Ruminococcaceae*_UCG-014, *Ruminococcus*_1 and *Turicibacter* were negatively correlated with the levels of FBG, HbA1c, AUC, Insulin, FI, WI, ALT, AST, BUN, TC, TG, LDL-c, TBA, IL-6, TNF-α, MDA and FFA and positively correlated with the levels of BW, SOD, GSH-Px, AA, PA, BA and TSCFA. In addition, the abundance of *Citrobacte* and *Pseudomonas* were found to have closely positive correlation with the levels of FBG, HbA1c, AUC, FI, WI, ALT, BUN, TC, TG, LDL, TBA, IL-6, TNF-α and FFA and negatively correlation with the levels of BW, SOD, AA, PA, BA and TSCFA. Furthermore, the visual network (Figure 7B) presented the osculating correlation between the bacteria abundance and biochemical parameters (|r| > 0.8, FDR adjusted *p* < 0.01). The information indicated that the relative abundance of *Romboutsia* and *Pseudomonas* was negatively correlated with the levels of HbA1c, AUC, BUN, TG, LDL-C, TBA and FFA and positively correlated with BW, AA and TSCFA. An obvious negative correlation between *Ruminococcaceae*_UCG-014 and the levels of TG, TBA and MDA was observed. *Ruminococcus*_1 was negatively correlated with LDL-C and FFA levels. *Citrobacter* and *Eubacterium_coprostanoligenes*_group were negatively correlated with FBG and Cr levels, respectively.

### 3.6. Effects of SFPs on Gene Expression at the mRNA Level in Diabetic Rats

**Figure 8:**
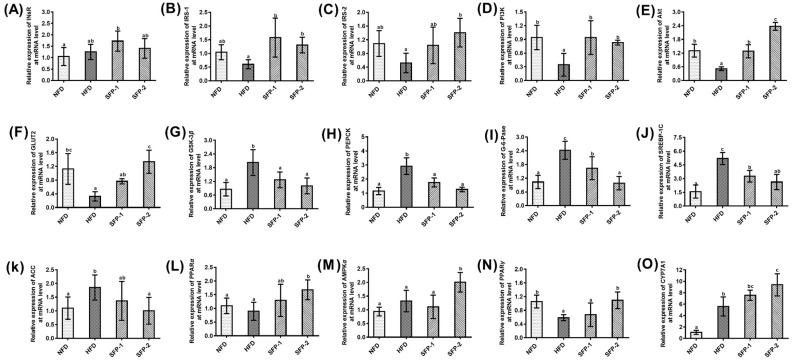
Effects of SFPs on expression of Ins R (**A**), IRS-1 (**B**), IRS-2 (**C**), PI3K (**D**), Akt (**E**), GLUT2 (**F**), GSK-3*β* (**G**), PEPCK (**H**), G-6-Pase (**I**), SREBP-1C (**J**), ACC (**K**), PPAR*α* (**L**), AMPK*α* (**M**), PPAR*γ* (**N**) and CYP7A1 (**O**) of liver in diabetic rats. The different letters represent significant difference (*p* < 0.05).

The key genes closely related to glucolipid metabolism, including Ins R, IRS-1, IRS-2, PI3K, Akt, GLUT2, GSK-3*β*, PEPCK, G-6-Pase, SREBP-1c, ACC, PPAR*α*, AMPK*α*, PPAR*γ* and CYP7A1, were investigated using RT-qPCR technology. The results are presented in Figure 8A–O. The gene expressions of PI3K, Akt, GLUT2 and PPAR*γ* were significantly lowered, and the expressions of GSK-3*β*, PEPCK, G-6-Pase, SREBP-1C, ACC and CYP7A1 were markedly increased in HFD group as compared with NFD group (*p* < 0.05). In comparison to HFD group, SFP treatment significantly up-regulated the mRNA relative expressions of IRS-1, PI3K and Akt and remarkably down-regulated the relative expressions of GSK-3*β*, PEPCK, G-6-Pase and SREBP-1C (*p* < 0.05). In particular, the regulatory effect of SFP-2 administration on Akt and G-6-Pase was greater than that of SFP-1 (*p* < 0.05). In addition, SFP-2 treatment also signally up-regulated the relative expressions of IRS-2, GLUT2, AMPK*α*, PPAR*γ* and CYP7A1 and down-regulated the ACC relative expression. These results indicate that SFPs could improve diabetes mellitus, possibly ascribed to the activation of the IRS/PI3K/AKT signaling pathway, inhibition of endogenous glucose production and acceleration of lipid metabolism. Notably, the effect of SFP-2 treatment on Akt, G-6-Pase IRS-2, GLUT2, AMPK*α*, PPAR*γ* and CYP7A1 was greater than that of SFP-1.

## 4. Discussion

Accumulating studies have proven that *Sargassum fusiforme* polysaccharides possess multiple pharmacological functions and have the potential be added to drugs or healthy foods for the adjuvant treatment of tumors, blood coagulation, oxidative stress, inflammation, etc. [20,26,27,28]. Our previous research also demonstrated that *Sargassum fusiforme* polysaccharides (SFPs) had better effects on regulating blood sugar and blood lipids after 5-week treatment in rats [21]. Nevertheless, the underlying anti-diabetic mechanisms of SFPs have not been studied. Therefore, in this study, diabetic rats were treated with SFPs for a longer period of time (8 weeks) to explore the hypoglycemic effects in rats. Ulteriorly, we investigated the possible mechanisms from several perspectives, such as inhibiting the blood glucose elevation caused by eating, remodeling intestinal flora, affecting blood glucose absorption and storage, and suppressing gluconeogenesis and lipid accumulation. The results indicated that SFPs could improve diabetic symptoms, hyperglycemia, insulin resistance, glucose tolerance, hyperlipemia, organ injury, chronic inflammation and oxidative stress, and SFP-2 showed better regulatory effects on the levels of TC, TG, LDL-C, FFA, TBA, BUN, SOD and MDA.

Digestive enzymes could break down carbohydrates in food, resulting in an increase in blood glucose level. Under normal physiological conditions, living organisms can quickly complete the absorption and storage of glucose in the blood, resulting in little change in blood glucose level [29]. When severe diabetes occurs, carbohydrate intake can cause greater blood glucose fluctuations. Accumulated research has shown that blood glucose fluctuations pose a greater threat to patient health than stable hyperglycemia [30]. As a consequence, inhibiting blood glucose fluctuation caused by eating is recognized as an effective therapeutic approach for diabetes treatment. The results of the present investigation revealed that SFP-2 could significantly reduce the blood glucose level in rats after starch loading; however, there was no significant difference in blood glucose level between the control group and SFP-2 group during glucose loading test, suggesting SFP-2 could restrain the postprandial blood glucose fluctuations by inhibiting carbohydrate hydrolase activity. Therefore, inhibition of digestive enzyme activity is one of the important approaches for SFP-2 to ameliorate diabetes mellitus.

Accumulating evidence has also indicated that gut flora composition is closely associated with host health [31]. The occurrence and development of many metabolic diseases, such as hyperlipidemia, chronic inflammation, diabetes and hepatic steatosis, are closely related to the compositional changes of intestinal flora [32]. Lipopolysaccharides (LPS) derived from the cell wall of bacteria might cause chronic inflammation, leading to insulin resistance and glycolipid metabolism disorders [33]. On the other hand, short chain fatty acids produced by microbial fermentation of difficult-to-digest carbohydrates could effectively regulate glucose and lipid metabolism disorders through balancing glucose absorption and storage and improving the functions of insulin target tissue [34]. Therefore, gut microbiota might improve diabetes mellitus via influencing LPS or SCAFs, and remodeling intestinal flora is an important approach to alleviate diabetes. Our previous research found that SFPs displayed anti-digestion properties under the simulated salivary, gastric and small intestinal conditions, and the current research showed that SFP-2 inhibited the activities of carbohydrate hydrolase in rat. These results suggest that SFPs may possess the potential to regulate intestinal flora, and the regulation of SFP-2 in intestinal flora may be greater than SFP-1. Fully understanding how SFPs influence intestinal flora is essential for understanding its antidiabetic mechanisms. The current results suggest that SFP administration could remarkably boost the growth of *Muribaculaceae_norank*, *Akkermansia*, *Bifidobacterium* and *Lactobacillus* in diabetic rats. In contrast, SFP-1 administration could increase the proportions of *Faecalibaculum* and *Ruminococcaceae*_NK4A214_group, while SFP-2 significantly increases the relative abundance of *Lachnospiraceae*_NK4A136_group, *Blautia*, *Olsenella* and *Romboutsia*. The correlation analysis showed that the relative abundance of these microorganisms was positively correlated with the improvement of diabetes-related indicators and the concentration of short-chain fatty acids. Similar to our results, accumulating studies have reported that the enrichment of *Lactobacillus* and *Bifidobacterium* could regulate the intestinal microenvironment and stimulate the growth of beneficial bacteria [16,35]. The growth of some short chain fatty acid producing bacteria, containing *Ruminococcaceae*_NK4A214_group, *Blautia*, *Olsenella*, *lachnospiraceae*_NK4A136_group, *Faecalibaculum* and *Romboutsia*, could reduce chronic inflammation, regulate immune function and improve glycolipid metabolism. The relative abundance of *Lactobacillus* and *Blautia* in the SFP-2 group was significantly higher than the SFP-1 group, which is consistent with our hypothesis. The above results show that intestinal flora regulation is one of the primary approaches for SFPs to improve diabetes.

Liver, a pivotal target organ of insulin, plays a crucial role in the maintenance of blood glucose and lipid homeostasis. Simultaneously, the liver is also an essential site for glycogen synthesis as well as gluconeogenesis [36]. To further evaluate the molecular mechanisms of SFP-1 and SFP-2 in improving diabetes mellitus in rats, gene expression closely related to glucose and lipid metabolism in the liver was measured by RT-qPCR. It is well established that the activation of the IRS/PI3K/AKT signaling pathway could accelerate the utilization of blood glucose in insulin target tissues [4]. The current results indicate that both SFP-1 and SFP-2 activated the IRS/PI3K/AKT signaling pathway and down-regulated the relative expressions of GSK-3*β*. In particular, SFP-2 administration also up-regulated GLUT2 expression, suggesting SFPs accelerated the absorption and storage of blood glucose by the liver. Gluconeogenesis is the process of transforming non-sugar substances such as glycerol, lactic acid and amino acids into glycogen or glucose [37]. When insulin resistance occurs, the dynamic balance of hepatic glucose production and glycogen synthesis is broken, and gluconeogenesis is increased, which further increases the blood glucose of patients [38]. Inhibiting the limiting velocity enzymes in gluconeogenesis expressions is an approach to reduce the occurrence of gluconeogenesis [39]. In comparison to the HFD group, SFP treatment significantly down-regulated the expressions of PEPCK and G-6-Pase. In addition, we also found that SFP-2 administration observably up-regulated the expressions of AMPK*α*, PPAR*γ* and CYP7A1 and down-regulated the relative expressions of ACC and SREBP-1C. Among them, the high expression of AMPK*α* is of great significance in improving insulin resistance and restoring islet cell function [40]. SREBP-1C is closely related to fatty acid metabolism and gluconeogenesis in the body [41]. Changes in gene expression of ACC, PPAR*γ* and CYP7A1 could affect fatty acid metabolism and insulin function in the liver [42]. The RT-qPCR analysis result showed that SFPs could improve insulin resistance and inhibit gluconeogenesis, and SFP-2 also could promote fatty acid oxidation and speed up cholesterol conversion into bile acids.

## 5. Conclusions

In summary, SFP administration could alleviate hyperglycemia, hyperlipemia, organ injury, chronic inflammation and oxidative stress. In particular, SFP-2 showed better regulatory effects on postprandial hyperglycemia, body weight, food intake and the levels of TC, TG, LDL-C, FFA, TBA, BUN, SOD and MDA in diabetic rats. SFP administration also could promote the growth of *Muribaculaceae_norank*, *Akkermansia*, *Bifidobacterium* and *Lactobacillus* in diabetic rats, and the abundance of *Lactobacillus* and *Blautia* in SFP-2 group was significantly higher than in the SFP-1 group. Additionally, SFP administration could regulate the genes involved in glycolipid metabolism in diabetic rats, and the effects of SFP-2 treatment on the relative expressions of Akt, G-6-Pase, IRS-2, GLUT2, AMPK*α*, PPAR*γ* and CYP7A1 were greater than those of SFP-1. Our future research will focus on the analysis of the fine structure of SFPs and the validation of the relationship between its gut microbiota regulation and hypoglycemic activity.

## Figures and Tables

**Table 1:** Primer sequences for real-time PCR.

Gene	Forward Primer	Reverse Primer
Ins R	CAATGGTGCTGAGGACACTAGG	GTGCTCTTCGTGGCTTGTGG
PI3K	GGTGAGGAACGAAGAATGGC	TCCGAGGCAAGACAGGGATA
Akt	TGAGACCGACACCAGGTATTTTG	GCTGAGTAGGAGAACTGGGGAAA
IRS-1	ATGTGGAAATGGCTCGGA	TAAGGCAGCAAAGGGTAGGC
IRS-2	TGACCAGTCCCACATCAGGC	CTGCACGGATGACCTTAGCG
GLUT2	CTCTGTGCTGCTTGTGGAGA	CGGCACAGAAAAACATGCC
GSK-3*β*	TCGTCCATCGATGTGTGGTC	TTGTCCAGGGGTGAGCTTTG
PEPCK	TGCCCATCGAAGGCATCA	TCTCATGGCAGCTCCTACAAACAC
G-6Pase	TCGTCCATCGATGTGTGGTC	TTGTCCAGGGGTGAGCTTTG
AMPK*α*	ACCTGAGAACGTCCTGCTTG	TAAGGCAGCAAAGGGTAGGC
PPAR*α*	TCACACAATGCAATCCGTTT	GGCCTTGACCTTGTTCATGT
PPAR*γ*	TGTCGGTTTCAGAAGTGCCTTG	TTCAGCTGGTCGATATCACTGGAG
ACC	GAGAGGGGTCAAGTCCTTC	ACATCCACTTCCACACACGA
SREBP-1C	TACTTCTTGTGGCCCGTACC	TCAGGTCATGTTGGAAACCA
CYP7A1	GCTTTACAGAGTGCTGGCCAA	CTGTCTAGTACCGGCAGGTCATT
*β*-actin	TGCTATGTTGCCCTAGACTTCG	GTTGGCATAGAGGTCTTTACGG

## Data Availability

Data is contained within the article.
